# New Tablet-Based Written Examination System for Metamorphopsia Quantification

**DOI:** 10.3390/jcm14061831

**Published:** 2025-03-08

**Authors:** Hisashi Fukuyama, Kazuma Mikami, Yoichi Okita, Eri Tahara, Yuki Yamamoto, Masataka Imura, Fumi Gomi

**Affiliations:** 1 Department of Ophthalmology, Hyogo Medical University, 1-1 Mukogawa-cho, Nishinomiya 663-8501, Japangomi.fumi@gmail.com (F.G.); 2 School of Science and Technology, Kwansei Gakuin University, Sanda 669-1330, Japan; kazuma446ra@gmail.com (K.M.); m.imura@kwansei.ac.jp (M.I.)

**Keywords:** metamorphopsia, central serous chorioretinopathy, epiretinal membrane, Amsler chart

## Abstract

**Background**: We aimed to develop a tablet-based written examination system (Implementing digitization in assessment for metamorphopsia: IDAM) to quantify metamorphopsia severity and monitor changes after treatment in patients with epiretinal membrane (ERM) and central serous chorioretinopathy (CSC). **Methods**: This prospective study included 33 eyes from 31 patients with ERM or CSC. Patients used a tablet and stylus to illustrate perceived line distortions with IDAM. IDAM displayed a grid at a size of 20° × 20° (771 × 771 pixels), and patients depicted any distortions that they perceived in the presented lines. Metamorphopsia scores were calculated by summing the line deviation distances. Scores and distortion areas were compared before and three months after treatment. **Results**: Thirty eyes had baseline metamorphopsia on IDAM. The average pretreatment IDAM scores were 196,598 pixels (ERM) and 98,414 pixels (CSC). IDAM and M-charts scores were correlated (r = 0.38, *p* = 0.03). IDAM scores improved post-treatment in both groups (both *p* < 0.001). Distortion areas decreased from 6.6 to 4.2 (*p* = 0.0049). **Conclusions**: IDAM allowed quantitative metamorphopsia evaluation and detected treatment responses. This tablet-based system could facilitate at-home monitoring in macular disorders.

## 1. Introduction

Metamorphopsia, characterized by the perception of wavy or distorted lines, is a prevalent visual distortion observed in various retinal disorders [1]. This symptom significantly affects the quality of vision [1,2,3]. Moreover, metamorphopsia often presents as an initial symptom or a recurring sign of retinal disorders [4]. Evaluating metamorphopsia could play an important role in the early detection and monitoring of retinal diseases [5,6].

Various methods have been proposed to detect metamorphopsia [4,7,8,9,10,11,12]. The Amsler chart has long been the gold standard for evaluating metamorphopsia. With its simple design, the Amsler chart is a widely accepted method. The limitations of the Amsler chart include this method lacking a precise and quantitative assessment of metamorphopsia severity, hindering the accurate monitoring of disease progression and treatment efficacy [10]. For evaluating metamorphopsia quantitatively, other approaches such as PreView PHP [11] and M-charts [12], both of which employ graded classification, are often used. Recently, some digital devices have been used for detecting metamorphopsia [13,14,15,16,17,18]. However, there is still some uncertainty as to whether these methods can accurately capture patients’ subjective visual symptoms.

To overcome these limitations, we have developed an original tablet-based written examination system (Implementing digitization in assessment for metamorphopsia: IDAM) for quantifying metamorphopsia. IDAM is a new test based on a digital tablet, on which patients write their own subjective visual symptoms. Our goal was to develop this method to provide an objective measurement of severity for metamorphopsia in patients with macular disease. The purpose of this study was to evaluate the efficacy of IDAM. We applied this new test to measure metamorphopsia in patients with epiretinal membrane (ERM) and central serous chorioretinopathy (CSC), evaluating postoperative changes quantitatively.

## 2. Materials and Methods

This is a prospective observational study. The Institutional Review Board of Hyogo Medical University approved this study (No. 2875), which followed the tenets of the Declaration of Helsinki.

Participants were recruited from the Department of Ophthalmology at Hyogo Medical University (Japan), and informed consent was obtained from all individual participants involved in the study. The investigational device, IDAM, was explicitly labeled for investigational use only and was used exclusively in the context of this research following the provision of informed consent. Study monitoring, data management, and record archiving were conducted in accordance with institutional and regulatory guidelines to ensure compliance and reliability.

### 2.1. Participants

This prospective study was conducted at the Department of Ophthalmology at the Hyogo Medical University (Japan) between July 2021 and April 2022. We recruited patients diagnosed with epiretinal membrane ERM or CSC at the same department, who were planning to undergo treatment. ERM was confirmed using optical coherence tomography (OCT). CSC was confirmed based on OCT findings showing serous subretinal fluid, fluorescein angiography (FA) detecting one or more leakage points, and the absence of macular neovascularization as determined by indocyanine green angiography or OCT-angiography. ERM was confirmed by optical coherence tomography (OCT). CSC was confirmed by OCT showing serous subretinal fluid and by fluorescein angiography (FA) detecting one or more leakage points, and the absence of macular neovascularization on indocyanine green angiography or OCT–angiography. The exclusion criteria for all subjects were the presence of other ocular diseases (age-related macular degeneration, optic neuritis, pathologic myopia, glaucoma, or a history of retinal disease) and patients who could not take part in the examination. We collected the following demographic information from each patient: age, sex, best corrected visual acuity (BCVA), spherical equivalent, OCT information, conventional Amsler grid test, and M-charts. M-chart scores were obtained for vertical and horizontal tests separately, and their mean value (mean M-chart score) was used for further data analysis.

Participants also completed a two-question usability survey compared to the conventional Amsler grid at baseline. The questionnaire items were as follows: (1) Which one expresses the metamorphopsia more accurately? (2) Which one more accurately identifies the location of the metamorphopsia?

For patients who had metamorphopsia at baseline visits, IDAM was also performed at month 3 after treatment.

### 2.2. Procedure

**Figure 1:**
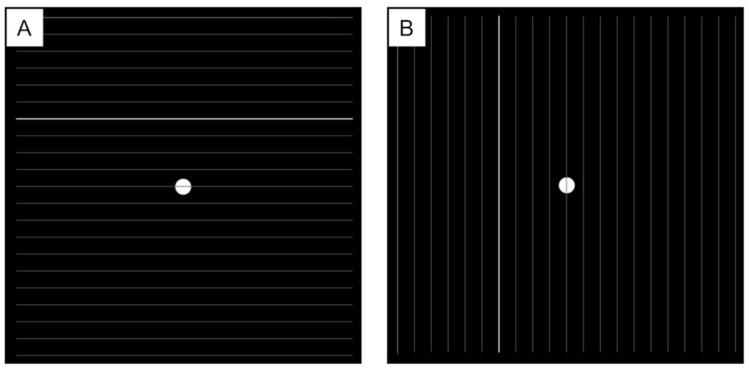
Display of the IDAM. Patients were instructed to stare at the center of the grid (white dot), and lines (highlighted white line) were sequentially displayed one at a time. The examination was conducted along both horizontal (**A**) and vertical lines (**B**).

**Figure 2:**
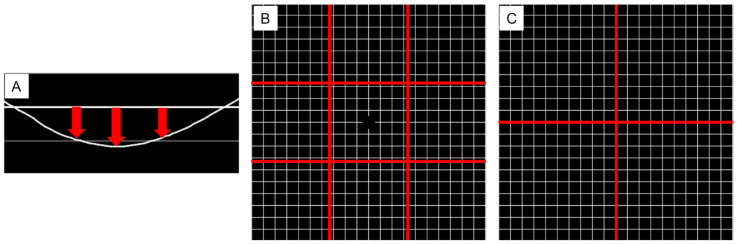
The total distance between the line drawn by the patient and the reference line (red arrows) was defined as the metamorphopsia score. (**A**) Metamorphopsia scores were calculated for all reference lines, and the sum of these distances was defined as the IDAM score. We also divided the display into nine regions (divided by red lines) and evaluated the presence or absence of metamorphopsia in each area. (**B**) Metamorphopsia scores were also calculated for both vertical and horizontal lines passing through the center. (red lines) (**C**).

The examination was conducted in a well-lit room using Microsoft’s Surface tablet (screen size, 12.3 inches; resolution, 2160 × 3840 pixels). Patients stared at the monitor at a distance of 30 cm with near vision correction; patients were presented monocularly. They were instructed to keep looking at the fixation point at the center of the monitor. The grid was displayed at a size of 20° × 20° (the total image dimensions were 771 pixels × 771 pixels, with a total pixel count of 594,441 pixels). This system utilizes a tablet interface to present lines to patients. One line at a time is sequentially displayed from above within the designated grid area. Next, the vertical lines are displayed from left to right. By presenting lines individually, this method aims to enhance the patient’s vision of their own visual image. The patient is then instructed to indicate whether there is any distortion in the presented line and, if so, to illustrate its appearance on the tablet. The patient utilizes a stylus pen with the tablet to depict their vision of the presented lines. To ensure accurate results, a patient needs to maintain fixation on the center of the screen and avoid unintentional shifts of gaze towards the grid area. To reduce the examination’s time, we showed a part of the lines in the grid first. If patients had metamorphopsia in each line, we checked other lines next to the line. The examination was conducted with both horizontal lines and vertical lines. To evaluate metamorphopsia quantitatively, we calculated the sum of distances between lines drawn by patients and the reference line (see Supplementary Video S1). We divided the test area into 9 sections and evaluated the value of the area detected in metamorphopsia (Figure 1). We defined this sum of distances as the metamorphopsia score in IDAM (IDAM score) (Figure 2).

### 2.3. Statistical Analysis

All statistical analyses were performed using JMP^®^ Pro (version 15.2.0, SAS Institute Inc., Cary, NC, USA). BCVA was expressed in a logarithm of the minimum angle of resolution (log MAR). Data were expressed as mean ± standard deviation. We used the Wilcoxon signed-rank test to compare BCVA and IDAM scores before and after treatment. The correlation between M-charts scores and IDAM scores were determined using the Spearman rank correlation coefficient. *p* < 0.05 was considered statistically significant.

## 3. Results

### 3.1. Baseline Characteristics

A total of 33 eyes in 31 patients were included in this study. We tested 11 eyes in 11 patients with ERM and 22 eyes in 20 patients with CSC before treatment. The baseline characteristics of patients are shown in Table 1. Metamorphopsia was detected on the M-chart in 28 eyes, and the average mean M-chart score was 0.62. The vertical M-chart score (MV) and horizontal M-chart score (MH) were 0.54 and 0.70, respectively.

### 3.2. IDAM Results

At baseline, 30 of the 33 eyes (ERM, 11; CSC, 19) had metamorphopsia on IDAM. The average IDAM score was 131,142 pixels (ERM, 196,598 pixels; CSC, 98,414 pixels). We extracted the metamorphopsia score for a vertical line and a horizontal line passing through the center point, which were 2664 pixels and 2349 pixels, respectively. The questionnaire results for patients’ preferences between the conventional Amsler grid and IDAM are presented in Table 2. For both question 1 (‘Which one expresses the metamorphopsia more accurately?’) and question 2 (‘Which one more accurately identifies the location of the metamorphopsia?’), 45.1% of the patients responded that IDAM was more accurate.

### 3.3. The Correlation Between M-Charts and IDAM

Of the 33 eyes, 27 showed metamorphopsia in both the M-charts and IDAM tests. The agreement rate of detection between IDAM and M-charts was 87.9% (29 eyes out of 33 eyes). Within the disagreement in four cases, in three cases, metamorphopsia was detected on IDAM only, and in one case, metamorphopsia was detected on M-charts only. Among the three cases where metamorphopsia was detected on IDAM only, two cases did not detect metamorphopsia on the center line. The average metamorphopsia score on IDAM was significantly correlated with the mean M-charts score (rs = 0.38, *p* = 0.03).

The metamorphopsia score for a vertical line on IDAM correlated significantly with MV (rs = 0.46, *p* = 0.007), and the score for a horizontal line significantly correlated with MH (rs = 0.40, *p* = 0.02).

### 3.4. Location of Metamorphopsia on IDAM

In a test area divided into nine sections, an average of 6.0 areas were detected in metamorphopsia. The ERM group had an average of 7.0 areas of metamorphopsia and 5.5 areas in the CSC group. Out of 30 eyes (ERM, 11 eyes; CSC, 19 eyes) that had metamorphopsia on IDAM, 23 eyes (76.6%) showed metamorphopsia in the central area (ERM, 10 eyes; CSC, 13 eyes).

### 3.5. Time Course of IDAM Results After Treatment

Among 33 eyes, 30 eyes (ERM, 11 eyes; CSC, 19 eyes) who had metamorphopsia on IDAM were also tested at month 3 after treatment. All of the ERM eyes received ERM and inner limiting membrane peeling. Of the 11 ERM eyes, 10 underwent cataract surgery. Among the CSC eyes, 16 of 19 received photodynamic therapy, and 3 of 19 received focal photocoagulation therapy. The preoperative and postoperative characteristics of eyes are shown in Table 3. The mean MV and MH values at 3 months (0.30 and 0.43, respectively) showed significant reductions from the baseline (*p* = 0.021 and *p* = 0.0068, respectively).

**Figure 3:**
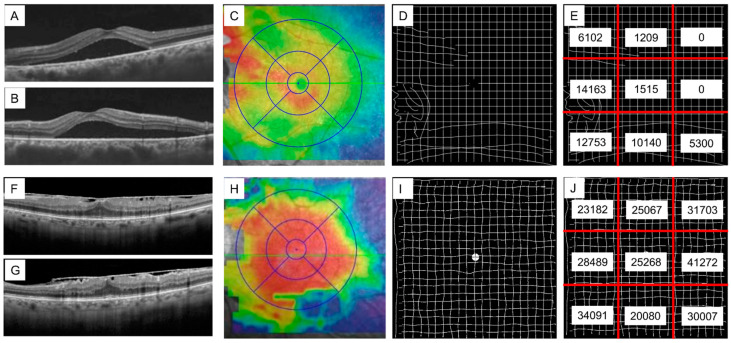
Horizontal B-scan OCT (**A**,**F**), vertical B-scan OCT (**B**,**G**), OCT MAP (**C**,**H**), IDAM (**D**,**I**), and IDAM scores in each of the nine sections in patients with epiretinal membrane (ERM) and central serous chorioretinopathy (CSC). Left eye of a 43-year-old male with CSC (**A**–**E**): OCT revealed subretinal fluid in the nasal area, extending to the macula. IDAM indicated distorted vision on the temporal side of the center, with an IDAM score of 51,182 pixels. Left eye of a 74-year-old female with ERM (**F**–**J**): OCT showed an epiretinal membrane in the macular area. IDAM revealed distorted vision across all nine areas, with an IDAM score of 259,159 pixels.

The area of metamorphopsia was significantly reduced after treatment, decreasing from 6.6 to 4.2 areas (*p* = 0.0049). After treatment, the average IDAM score was reduced from 144,256 pixels to 67,163 pixels. (*p* < 0.001) (Figure 3).

## 4. Discussion

In this study, we present a new tablet-based method, IDAM, for quantifying metamorphopsia severity and location patterns in patients with retinal diseases. A key advantage of IDAM is the ability for patients to illustrate their own visual distortions directly on a digital interface. This allows the evaluation of the quantitative assessment of metamorphopsia, including location and severity. Furthermore, IDAM allows us to monitor improvement following treatment.

The Amsler chart is a common method for detecting metamorphopsia. This chart utilizes a grid pattern, allowing patients to identify areas of distortion or missing vision [4]. It is suitable for home use, enabling the self-monitoring of central vision. To utilize digital platforms for monitoring, several digital applications based on Amsler grid have been proposed [13,14,15,16,17,18]. In comparison with these applications, IDAM stands out due to its unique features allowing patients to input their own visual symptoms. This is a key strength compared to prior metamorphopsia tests relying on examiners decoding patient tracings. Previously, Shinoda et al. introduced a modified Amsler chart, requiring patients to trace irregular or curved grid lines on paper [8]. Our methodology employs the computer-assisted analysis of patient renderings without external translation. IDAM enables patient self-documentation and the evaluation of metamorphopsia patterns directly from visualized images. The direct symptom illustration and computational quantification make IDAM a more patient-centric and sensitive technique for assessing this complex visual complaint. Our usability survey revealed mixed preferences. While nearly one third of patients favored the conventional Amsler chart, almost half preferred IDAM for its accuracy in expressing and identifying the location of metamorphopsia. This highlights the potential of IDAM as a valuable alternative for personalized and precise assessment.

IDAM detected metamorphopsia within a 20-degree visual field and provided information on the locations of metamorphopsia. In this study, patients reported metamorphopsia in 6.0 areas on average. In addition, 68% of patients who had metamorphopsia reported metamorphopsia outside central areas. This study revealed that patients reported experiencing metamorphopsia in areas outside the central region, showing IDAM’s capability to detect distortions beyond the central field.

IDAM enables the quantitative assessment of metamorphopsia. In this study, the IDAM score significantly improved after treatment (from 144,256 to 67,163 pixels, *p* < 0.001). The number of areas detecting distortions also decreased after treatment (from 6.6 to 4.2 areas, *p* = 0.0049). Furthermore, the IDAM score correlated significantly with the M-chart score (rs = 0.38, *p* = 0.03). Our study showed that IDAM allows the quantitative evaluation and monitoring of metamorphopsia improvement after treatment.

The comparison between M-charts and IDAM for metamorphopsia detection in this study yielded an agreement rate of 87.9%. Compared to M-charts, two cases where M-charts failed to detect metamorphopsia were identified by IDAM along lines other than those passing through the central area. We previously reported that the Amsler chart detected metamorphopsia in retinal disorders, and the Amsler grid was more sensitive, especially for CSC. Furthermore, some patients had metamorphopsia outside the Amsler grid’s center lines in eyes with CSC [19]. In this study, our usability questionnaire revealed that while 60% of CSC patients preferred IDAM, only 18.2% of ERM patients favored it for its accuracy in detecting metamorphopsia. Based on this and previous results, IDAM may be superior in detecting extrafoveal diseases like CSC.

Our study has several limitations. Firstly, the limitations of our study include a relatively small sample size. Secondly, our study focused on eyes with ERM and CSC, excluding conditions such as age-related macular degeneration, macular holes, and rhegmatogenous retinal detachment, all of which can also cause metamorphopsia. Thirdly, we did not confirm the repeatability of the examinations, and establishing a definitive ground truth for metamorphopsia is challenging due to its subjective symptoms. Future studies should prioritize evaluating the repeatability of the tests. Fourth, we did not evaluate the association between macular morphology and IDAM. When using IDAM on monitoring, it is crucial to observe the progression and reproducibility in eyes with macular disease, as well as its correlation with macular morphology. Fifth, our study did not include long-term follow-up. Lastly, the design of this study had selection bias. Our study included patients who could follow the operations of our system. However, for effective monitoring, operational improvements are needed, particularly for older and low-vision patients. For better monitoring, we need to improve operation. Additionally, patients might shift their fixation point during the procedure, which could be addressed by incorporating an eye-tracking device into the new system to confirm fixation on the center during responses. Nevertheless, IDAM has the potential to quantitatively and accurately capture metamorphopsia, including localization. This suggests its potential utility in at-home monitoring for retinal diseases.

## 5. Conclusions

A new tablet-based method, IDAM, has been developed to quantify the severity and location patterns of metamorphopsia in patients with retinal diseases. This method is particularly useful in assessing and localizing metamorphopsia and monitoring its severity. Additionally, IDAM enables tracking improvements following treatment. Future studies are needed to evaluate its efficacy as a screening and monitoring tool for eyes with macular diseases.

## Figures and Tables

**Table 1:** Patients’ baseline demographic and clinical characteristics.

	Total	ERM	CSC
Number of patients	31	11	20
Number of eyes	33	11	22
Age (years)	59.0 ± 13.2	69.8 ± 8.6	53.0 ± 11.5
Gender (male) (%)	24 (77.4%)	7 (63.6%)	17 (85.0%)
Eye (right) (%)	17 (51.5%)	4 (36.4%)	12 (54.5%)
Log MAR BCVA	0.142 ± 0.243	0.299 ± 0.247	0.064 ± 0.204
Equivalent square	−1.5 ± 2.9	−3.0 ± 3.1	−0.82 ± 2.6
M-charts (vertical)	0.54 ± 0.59	1.12 ± 0.66	0.25 ± 0.25
M-charts (horizontal)	0.70 ± 0.61	0.89 ± 0.65	0.60 ± 0.58

ERM, epiretinal membrane; CSC, central serous chorioretinopathy; log MAR BCVA, logarithm of the minimum angle of resolution for best-corrected visual acuity.

**Table 2:** Questionnaire results.

Questions	Answers	Total (*n* = 31)	ERM (*n* = 11)	CSC (*n* = 20)
Which method describes the metamorphopsia most accurately?	Amsler	11 (35.1%)	7 (63.6%)	4 (20.0%)
	IDAM	14 (45.1%)	2 (18.2%)	12 (60.0%)
	Neutral	6 (19.4%)	2 (18.2%)	4 (20.0%)
Which method most accurately identifies the location of the metamorphopsia?	Amsler	10 (32,3%)	7 (63.6%)	3 (15.0%)
	IDAM	14 (45.1%)	4 (36.4%)	10 (50.0%)
	Neutral	7 (22.6%)	0 (0%)	7 (35.0%)

ERM, epiretinal membrane; CSC, central serous chorioretinopathy.

**Table 3:** Clinical characteristics in eyes with metamorphopsia detected with IDAM.

	Total	ERM	CSC
Number of eyes	30	11	19
Log MAR BCVA			
Baseline	0.138 ± 0.253	0.299 ± 0.247	0.045 ± 0.211
Postoperative	0.004 ± 0.194	0.144 ± 0.221	−0.076 ± 0.120
Equivalent square			
Baseline	−1.7 ± 3.0	−3.0 ± 3.1	−0.88 ± 2.7
Postoperative	−1.4 ± 2.5	−1.75 ± 1.9	−1.12 ± 2.8
M-charts (vertical)			
Baseline	0.57 ± 0.61	1.12 ± 0.66	0.25 ± 0.23
Postoperative	0.30 ± 0.42	0.56 ± 0.51	0.15 ± 0.51
M-charts (horizontal)			
Baseline	0.75 ± 0.61	0.89 ± 0.65	0.67 ± 0.59
Postoperative	0.43 ± 0.55	0.47 ± 0.66	0.40 ± 0.49
CFT (µm)			
Baseline	353 ± 146	425 ± 167	313 ± 120
Postoperative	242 ± 107	350 ± 95	181 ± 49

ERM, epiretinal membrane; CSC, central serous chorioretinopathy; log MAR BCVA, logarithm of the minimum angle of resolution for best-corrected visual acuity; CFT, central foveal thickness.

## Data Availability

Data will be made available upon reasonable request.

## Supplementary Materials

The following supporting information can be downloaded at: https://www.mdpi.com/article/10.3390/jcm14061831/s1. Video S1. The procedure of IDAM.
